# Social network analysis of multi-stakeholder platforms in agricultural research for development: Opportunities and constraints for innovation and scaling

**DOI:** 10.1371/journal.pone.0169634

**Published:** 2017-02-06

**Authors:** Frans Hermans, Murat Sartas, Boudy van Schagen, Piet van Asten, Marc Schut

**Affiliations:** 1 Leibniz Institute for Agricultural Development in Transition Economies (IAMO), Theodor-Lieser-Strasse 2, Halle (Saale), Germany; 2 Knowledge, Technology and Innovation Group, Wageningen University, EW Wageningen, The Netherlands; 3 International Institute of Tropical Agriculture (IITA), Kacyiru, Kigali, Rwanda; 4 Swedish University of Agricultural Sciences (SLU), Department of Urban and Rural Development, Ulls väg Uppsala, Sweden; 5 Bioversity International, Quartier Kabondo, Rohero 1, Avenue 18 Septembre 10, Bujumbura, Burundi; 6 International Institute of Tropical Agriculture (IITA), Kampala, Uganda; Utrecht University, NETHERLANDS

## Abstract

Multi-stakeholder platforms (MSPs) are seen as a promising vehicle to achieve agricultural development impacts. By increasing collaboration, exchange of knowledge and influence mediation among farmers, researchers and other stakeholders, MSPs supposedly enhance their ‘capacity to innovate’ and contribute to the ‘scaling of innovations’. The objective of this paper is to explore the capacity to innovate and scaling potential of three MSPs in Burundi, Rwanda and the South Kivu province located in the eastern part of Democratic Republic of Congo (DRC). In order to do this, we apply Social Network Analysis and Exponential Random Graph Modelling (ERGM) to investigate the structural properties of the collaborative, knowledge exchange and influence networks of these MSPs and compared them against value propositions derived from the innovation network literature. Results demonstrate a number of mismatches between collaboration, knowledge exchange and influence networks for effective innovation and scaling processes in all three countries: NGOs and private sector are respectively over- and under-represented in the MSP networks. Linkages between local and higher levels are weak, and influential organisations (e.g., high-level government actors) are often not part of the MSP or are not actively linked to by other organisations. Organisations with a central position in the knowledge network are more sought out for collaboration. The scaling of innovations is primarily between the same type of organisations across different administrative levels, but not between different types of organisations. The results illustrate the potential of Social Network Analysis and ERGMs to identify the strengths and limitations of MSPs in terms of achieving development impacts.

## Introduction

Multi-stakeholder platforms (MSPs) are increasingly seen as promising vehicles for agricultural innovation and development [1, 2]. In the field of agricultural research for development, MSPs are expected to contribute to a structural and long-term engagement among stakeholders for overcoming complex agricultural problems [3]. Key characteristics of complex problems in agricultural systems are their multiple dimensions (biophysical, technological, socio-cultural, economic, institutional and political), and their embeddedness across different scales, hierarchical levels and interdependent actors. As a result, complex problems possess inherent uncertainties that defy prediction and linear innovation pathways [4–6]. They often are a mix of socio-political issues where different world views, norms and values collide with different interests. Consequently, proposed solutions in different scenarios can result in turning different stakeholders into winners or losers.

The continuous engagement of various stakeholders in exploring innovations to address these complex agricultural problems is essential for three reasons. First, stakeholder groups can provide various complementary insights about the biophysical, technological and institutional dimensions of the problem, broadening the knowledge base. By engaging in a social learning process with each other, stakeholders can negotiate what type of innovations are technically feasible, economically viable, and social-culturally and politically acceptable [4, 7, 8]. Second, through their interaction and participation, stakeholder groups become aware of their different interests, needs and objectives, but also of their fundamental interdependencies and the need for concerted action at different levels to overcome their constraints and reach their objectives [9–11]. Third, stakeholders are more likely to accept or support the implementation of innovations when they have been part of its development process [12, 13].

Multi-stakeholder approaches, including MSPs, can therefore play an important role in facilitating innovation to overcome complex problems and achieving development impacts [14, 15]. Two key objectives for working with MSPs for agricultural development are (1) to enhance ‘capacity to innovate’ in stakeholder networks, and (2) to contribute to the scaling of innovations to achieve development impacts [16]. Over the past 5–10 years, there has been increasing enthusiasm and optimism on the role of MSPs for agricultural innovation and scaling in developing countries. Consequently, MSPs have been implemented on a case-by-case basis at selected sites. However, very little evidence has been systematically collected on whether and how MSPs actually support functions that can foster innovation and scaling.

The objectives of this paper are to explore (i) the capacity to innovate and (ii) the potential for scaling of innovations of three MSPs situated in the different governance contexts of Burundi, Rwanda and the South Kivu Province located in eastern Democratic Republic of Congo (in the remainder of this paper referred to as DRC for short). To achieve these objectives we will study these MSPs from a network perspective by analysing the linkages between different types of stakeholder organisations and how the structure of these linkages inhibits or facilitates innovation and scaling of innovation.

### Key-concepts

Innovation is defined as the successful combinations of ‘hardware’, ‘software’ and ‘orgware’ that have been implemented and brought into use to serve a specific public or private purpose [17, 18]. In this view innovations not only require new technologies or tools (‘hardware’), but also new knowledge, processes and new modes of thinking (‘software’) and a reordering of institutions and of organisations (‘orgware’). Innovations thus emerge from the complex interactions among a diverse set of public, private and civil society actors engaged in generating, exchanging and using knowledge within a so-called Agricultural Innovation System (AIS) [19, 20]. The AIS framework has broadened views of agricultural innovation processes in two important ways. First, it has recognized that actors beyond those directly involved in the agricultural production chain and the agricultural research, extension and education system play a role in innovation processes (e.g. service providers, financial sector, civil society). Second, it stresses the importance of the constraining and enabling influence of institutions (defined as the ‘formal and informal rules of the game’) in innovation processes [21–23].

Within this AIS framework, the capacity to innovate is defined as the ability of different groups of stakeholders to continuously identify and prioritize problems and opportunities in the dynamic environment that they are in, and take risks and experiment with different new combinations of technical and institutional configurations and assess the trade-offs from these options [24]. Within an AIS framework the scaling of an innovation refers not only to the successful adaptation and adoption of technologies but also includes the successful implementation of new institutional arrangements to expand their impact [25, 26]. Two different types of scaling are relevant in this regard. Outscaling refers to the horizontal diffusion process of innovations among organisations at the same administrative level (e.g. within district, provincial, national or supranational levels). This is more or less similar to the classic (technology) adoption and diffusion model of Rogers [27]. Upscaling of an innovation refers to the institutional uptake or embedding of processes or technologies by organisations at higher administrative levels (e.g. across district, provincial, national or supranational levels). This process requires institutional entrepreneurship and political influence to change rules and regulations [28–30].

Collaboration among stakeholders is a central element of the MSP approach that seeks to enhance both the capacity to innovate and the scaling of an innovation. The capacity to innovate benefits from the interaction between a variety of stakeholders with access to different sources of knowledge and power that can strengthen their collective agency [4, 31, 32]. At the same time, collaboration connects actors and organisations within and across different administrative levels which is important for out- and upscaling [33, 34].

In this paper we take a network perspective on collaboration which means that we focus on the structure of the relationships between collaborating partners within an MSP. Collaboration processes are essentially relational in nature: they require the creation and maintenance of a connection between one or more actors or organisations. In AIS, understanding the changes in collaboration resulting from interventions such as a MSP requires monitoring changes in stakeholder networks [19]. Given the relational nature of MSP activities, Social Network Analysis (SNA) offers a framework to study and model different aspects of agricultural innovation and scaling [35, 36]. SNA can enable a better understanding of the complexity and multi-dimensionality of multi-stakeholder innovation processes [37, 38].

The central question of this paper is: What relational pattern of collaborative ties in a MSP fosters (1) the capacity to innovate and (2) the scaling of innovations? We will answer this question by comparing the collaborative networks of the MSPs in three countries in Central Africa: Burundi, Rwanda and DRC. As these MSPs were relatively ‘young’ (approximately 1 year at time of data collection), we assess their innovation and scaling potential, rather than their performance. We focus specifically on collaborative ties between organisations because those ties are also the conduits for knowledge exchange and influencing that are crucial for innovation and scaling processes. Based on our analysis of the innovation literature we propose that:

1. Capacity to innovate requires:
  - Broad, multidisciplinary networks with a diversity of stakeholders from business, government, civil society and knowledge institutes who contribute to effective social learning processes, identify and analyse complex problems and explore innovations to address them [20, 39–42].
  - The availability of powerful and influential persons or organisations within the network that can support agenda setting, mobilize resources, provide legitimacy and a mandate to create space (or niches) for innovation, and counteract resistance to change [9, 43–45].
2. The potential for upscaling and outscaling of innovations depends on the characteristics of the collaborative network. More specifically:
  - Dense collaborative networks facilitate the exchange and dissemination of information.
  - To ensure upscaling, organisations at different administrative levels have to be connected to each other, so that information and other resources can flow easily across different levels.

In Table 1 these network characteristics have been summarized. It has to be noted that these network characteristics are formulated fairly broadly and we do not want to suggest that there is an optimum network configuration that maximizes the innovation or scaling potential in all governance contexts. A diverse group of stakeholders who draw on different sources of knowledge is important to solve complex problems, but when the cognitive distance between actors becomes too large it becomes difficult to establish common ground [46, 47]. Similarly, there comes a point when the density of a network can become problematic. Isaac [40], for example, explains how knowledge networks with high density may result in collective action (essential for scaling) but little new information (essential for innovation), whereas a low density network may invite new information (essential for innovation) but the exchange of such information may be impeded (essential for scaling). Powerful actors in the network can facilitate change, but they are also likely to be invested in the status quo and therefore can stifle innovations that threaten their power base [48].

**Table 1:** Network characteristics to evaluate capacity to innovate and scaling potential of MSPs.

Network objective	Network process	
	Knowledge exchange	Influence
1. Capacity to innovate	1a. Broad networks with multidisciplinary partners enhance social learning	1b. Centrality of influential organisations within the network facilitates institutional entrepreneurship, agenda setting and creation of space for experimentation
2. Scaling of innovation	2a. Dense collaborative networks facilitate the exchange and dissemination of information (outscaling)	2b. Multi-level networks facilitate the institutionalisation of an innovation (upscaling).

It is clear, therefore, that the effectiveness of an innovation network depends on the context and the actors involved. In this regard it is helpful to think about these characteristics as the opposite of some well-known innovation failures: sparse, disconnected innovation networks represent a barrier, or a systemic innovation failure [21], and without influential organisations present in the network no changes can be made at all. Furthermore, we assume that within the context of Central Africa, the lack of innovation capacity and the resulting innovation failures have to do with a dearth of linkages between organisations and coordination efforts across scales, and as such the MSP have been established with the particular aim to remedy some of these network failures [49, 50].

Based on the innovation network characteristics in Table 1, we can identify a number of social processes at the micro level that might result in empirically observable innovation networks with high capacity to innovate and high scaling potential. We assume that when ‘opposites attract’, (a process that is in the network literature is also referred to as heterophily), it will result in a broad and diverse network connecting different types of organisations. With regard to the capacity to innovate we hypothesize that in MSPs:

1 Organisations (e.g. farmer, government, NGO, business, research) tend to form more network links with different types of organisation.
2 Organisations that are perceived as being influential will be preferred collaboration partners for stakeholders in MSPs. Therefore, influential partners should end up in more central positions in the collaborative network.

To ensure processes of outscaling and upscaling, information and knowledge has to flow among organisations located within and across different levels. With regard to scaling we can hypothesize that:

3 Information travels easier in denser innovation networks, which is beneficial for scaling.
4 Organisations with knowledge will make for more attractive collaboration partners.
5 Organisations tend to form more network links across administrative levels as compared to organisations operating at the same level (local, provincial, national or supranational).

In the remainder of this paper we will use these five hypotheses to identify and explain the characteristics, similarities and differences of the MSP networks in Burundi, DRC and Rwanda.

## Methodology

### Study sites

Data for this study were gathered within the framework the CGIAR Research Program on Integrated Systems for the Humid Tropics (Humidtropics) that has adopted the MSP approach for achieving its development outcomes. Research for Development Platforms and Innovation Platforms are core tools of the Humidtopics intervention strategy to bring together relevant actor groups and organisations, and to stimulate working together towards the realization of development outcomes. The collaboration between Innovation Platforms and Research for Development Platforms is expected to facilitate awareness about local innovations (tested in Innovation Platforms) at the (sub-)national level (in the Research for Development Platforms), which can stimulate that lessons and innovations can go to scale.

Data were collected with three Research for Development Platforms in the Action Sites of Burundi, DRC and Rwanda. Note that the DRC study site is about double the area but half as populous as those of Rwanda and Burundi. The selection of these three sites is based on several interesting similarities and differences when it comes to agricultural innovation [51]. Similarities are related to key agro-ecological and demographic features and agricultural productivity challenges. In general, the region is densely populated; agricultural pressure on land is high, and farm sizes are small (<2 ha) [52]. In these highly populated areas, soil fertility is one of the main constraints of agricultural production, driven by (i) absence of nutrient inputs, (ii) soil erosion and (iii) sub-optimal agricultural practices [49, 53]. Differences can be found in the governance context with the position and role of the government being different in the three countries: DRC (very decentralised) and Rwanda (very centralised) forming the two ends of the spectrum. As a result there are also differences in the effectiveness of public governance (i.e. formulation and implementation of policy), which is generally perceived as low in eastern DRC, medium in Burundi, and high in Rwanda [54, 55].

### Data collection, cleaning and analysis

Humidtropics initiated MSPs for innovation and scaling based on three types of stakeholder mappings. The first approach was to identify long-term established partners of the CGIAR centres in Burundi, eastern DRC and Rwanda. The second approach was participatory stakeholder mapping based on Humidtropics workshops for which potential partners were invited. The third approach was to prepare dissemination materials about the program and distribute them in different locations [56] to encourage organisations to join the MSP. As such, different types of organisations were provided the opportunity to form part of the MSP.

Data gathering for the network analysis was done during MSP meetings in Burundi, Rwanda and DRC in August 2014. Data were gathered during a regular MSP meeting where ongoing research and development activities are being discussed among the participants. Data collection was done by the authors and was harmonised across the three countries by using a detailed protocol. A name generator (open nomination) was employed based on the following question: “Compose a list of the names of all organisations with whom you collaborate”. In subsequent steps respondents were asked to identify within their initial list the 5 organisations that they viewed the most important for knowledge exchange (question asked was: “go through the list and circle the 5 organisations in the list that are most important for knowledge exchange”), and the 5 organisations that they viewed to be the most influential (question asked was: “Go through the list again and now underline the 5 organisations that you think are most influential”). In addition, the following information was collected: 1) name, gender, age and (multiple) affiliation(s) (questions asked were: “Write your name on the sheet of paper”, “Indicate your gender and age” and “Write down ALL the names of organisations/ companies/ institutes etc. that you represent”). The data collected therefore represent a ‘one-wave snowball sample’ of the platform. Since the data were gathered at the same meeting, there is some overlap between the organisations mentioned by the participants: some of the named ‘alters’ also appear on the ‘ego’ list. For Burundi, the overlap between egos and alters was 7.0%, for DRC 7.8% and for Rwanda 5.6%.

In total, 45 respondents representing three MSPs contributed to data gathering (Table 2). Average age of the respondents was approximately 43 years. 78% of the respondents were male.

**Table 2:** Characteristics of respondents (M = male, F = female).

Country	Respondents	Average age	Gender		Total distinct affiliations
			M	F	
Burundi	14	42	10	4	15
DRC	21	43	16	5	35
Rwanda	10	43	9	1	7
**Total**	**45**	**43**	**35**	**10**	

Data were entered and cleaned by the authors. Local MSP Facilitators supported the authors in matching of organisational abbreviations and full names, French versus English abbreviations and organisation names, deciphering handwriting and misspelling of organisation names and abbreviations. Furthermore, the MSP Facilitators provided additional information on the type of organisation (farmer organisation, NGO/ civil society, private sector, government, research and training) and the principle administrative level at which these organisations are active (supranational, national, provincial, district). The resulting networks are provided as csv- files in the S1 File, that also includes the organisations’ names, abbreviations levels and typology. Our participatory observations in the MSPs analysed in this paper enabled us to interpret the data and results.

### Social network analysis and Exponential Random Graph Models

We have applied exploratory social network analysis [57, 58] in combination with Exponential Random Graph Models (ERGMs). ERGMs belong to the class of statistical inference models and are among the most popular and theoretically well-developed class of network models [59]. ERGMs are used for testing hypotheses about the social processes that might have led to the creation and development of an empirically observed network. The statistics in these models are based on the occurrence of certain micro-level patterns of ties that indicate specific mechanisms of tie formation at work. Examples are preferential attachment (to popular nodes), reciprocity between nodes (resulting in the formation of a double arrow), transitivity (friends of friends are likely to become friends) resulting in a local triangle structure and processes of homophily in which two nodes with the same trait are more likely to form a tie. ERGMs are used to test statistically whether the relative occurrence of such patterns is consistent with these underlying dynamic processes of network formation. For a more detailed introduction into ERGMs see Lusher et al., Harris, and Lubell et al. [60–62]. The analysis of network properties and ERGM specification was done in R, using the statistical ‘statnet’ package (version 2016.9) [63], and the associated ‘ergm.ego’ package (version 0.3.0) [64]. See S1 R–scripts for an overview of the used analysis code. The ‘ergm.ego’ package was developed especially for ego-networks. In such ego-networks the collected data are considered to be a sample of a larger network of a known, or unknown size. In our case we did not know the total size of the population network of the AIS in all the three countries. In addition, the membership of the MSP is not fixed: it changes over time and the sample is therefore necessarily only capturing a snapshot picture of the ego-networks of MSP participants of what–in reality–is a dynamic process of collaboration and partnering. Nevertheless, the sample represents a reliable picture of the typical ego-networks at the national levels in Burundi and Rwandan and provincial level for DRC and as such can be used as input for ego network modelling.

The ergm.ego package is based on the finding by Krivitsky et al. [65] that it is possible to obtain a “per capita” size invariant parameterization for dyad-independent statistics by using an offset that preserves the mean degree (approximately equal to −log(n), where n is the number of nodes in the network). Simulations have suggested this is also possible for some dyad-dependent statistics. However, the processes of so-called ‘network self-organisation’ at the level of the entire network (like triadic closure, degree assortativity and 4-cycles) are not incorporated in the ergm.ego package. In their description of the package, Krivitsky and Morris [66] state that if the population network is not overly large the parametrization of such higher order effects might not be necessary.

Terms were added in consecutive blocks (node level and dyad level) to examine their relative contribution to enhancing the goodness-of-fit of the models [67]. Three models were tested and evaluated: starting with a simple random graph model (M0) (where all nodes have an equal chance to form a tie), and adding complexity in subsequent models by adding terms corresponding to our hypotheses at the node level (M1) and the dyad level (M2). We have scaled all our results to a”pseudo-population” size of 1,000 for all three countries, following the advice of Krivitsky and Morris [66].

At the node level we look at the degree (amount of ties) that organisations have within the knowledge network to test hypothesis 3. The knowledge degree serves therefore as an indication for the perception of other actors that an organisation possesses complementary knowledge. Following the AIS perspective we assume that such relevant knowledge is not limited to research and extension organisations, but is also possessed by farmers, NGOs, businesses, etc. To operationalise hypothesis 2 we take the indegree of organisations in the influence network as a measure of their perceived power. Again: not only government organisations are deemed to be powerful, but other types of organisations can also possess other forms of power [68]. At the dyad level we look at ties between different types of organisations. A typology was made in 6 different categories of actors: 1) business, 2) farmer, 3) government, 4) non-governmental organisations (NGOs), 5) research, training and extension, and 6) unknown. Hypothesis 1 is thus tested by looking at the tendency for different types of organisations to form collaborative ties. Finally, for scaling we look at the administrative level where organisations are (most) active: 1) local, 2) provincial, 3) national, 4 supranational, or 5) unknown. Hypothesis 4 is tested by investigating the tendency of actors working at different levels to form collaborative ties.

Models were checked for potential degeneracy (see S2 File) and goodness-of fit through visual inspection of the standard plots that the statnet package generates for this purpose, as suggested by Hunter et al. [69]. Since all the models underestimated the number of organisations with a degree of 1, we fixed this amount in the models to increase the fit. To ease the comparison of the plots, we have calculated a goodness-of-fit percentage following the example of Harris et al. [70]. The calculated percentage is based on the proportion of the relevant degree distribution that fall within the 95% confidence intervals of simulations based on the models. The term relevant here is not defined for all degrees, but only those degrees where either the results of the ergm.ego model, or the original measurement show a value unequal to 0.

## Results

### Descriptive network characteristics

**Fig 1:**
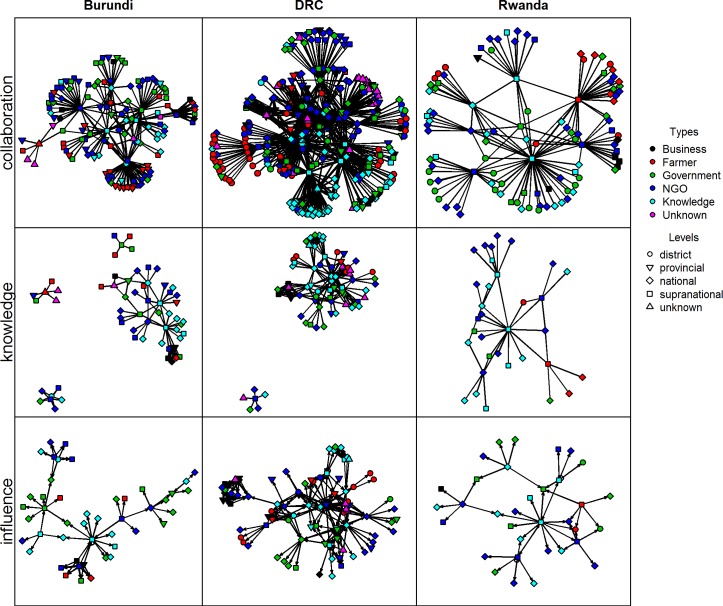
Overview of MSP networks for collaboration, knowledge exchange and perceived influence.

Due to the slight overlap in egos and alters in each country, we can depict the different networks in each country as if they are complete networks. We have thus constructed three networks for each country (Fig 1): a collaborative network based on organisational ties (first row), a knowledge exchange network (second row) and an influence network (third row). Even though ego-networks are typically directed, we have defined collaboration and knowledge exchange as a mutual relationship. These networks were therefore defined as undirected networks. No loops were allowed in these networks which implies that a respondent cannot exchange knowledge or collaborate with his or her own organisation. Influence was defined as a directed network and respondents were allowed to consider their organisation as influential, thus including loops in the networks. Below we will describe these networks and their implications for the capacity to innovate and scaling in more detail.

#### Collaborative networks

The collaborative network is smallest in Rwanda and largest in DRC (Table 3). In all three countries, the networks are dominated by NGOs in terms of composition. In Burundi and Rwanda, government organisations rank second. In DRC the second place is taken by research and training organisations (21%), but the difference with government organisations (18%) is rather small. Almost absent in all three countries are private sector organisations.

**Table 3:** Collaborative network composition and characteristics.

	Farmer organisations	NGO	Private sector	Government	Research and training	Unknown	Total nodes (g)	Total ties (L)
Burundi	27(19%)	51(36%)	8(6%)	32(23%)	19 (13%)	5 (5%)	142 (100%)	237
DRC	45 (16%)	82 (29%)	24 (9%)	50 (18%)	59 (21%)	20 (7%)	280 (100%)	903
Rwanda	14 (13%)	36 (33%)	6 (6%)	32 (30%)	20(19%)	0 (0%)	108 (100%)	142

In Table 4, the collaborative networks are broken down according to the administrative level that the organisations operate at. In the collaborative networks in Rwanda and DRC, the majority of organisations operate at the supranational level. In Burundi, the national level is the best represented. In Rwanda and Burundi some levels are missing in the network. In Rwanda, the provincial level is almost completely absent and in Burundi the district level is almost completely absent.

**Table 4:** Number and (percentage) of organisations per level in the collaborative network.

	District	Provincial	National	Supranational	Unknown	Total organisations
**Burundi**	3 (2%)	33 (23%)	57 (40%)	45 (32%)	4 (3%)	142 (100%)
**DRC**	65 (23%)	57 (20%)	36 (13%)	101 (36%)	21 (8%)	280(100%)
**Rwanda**	24 (22%)	1(1%)	23 (21%)	60 (56%)	0 (0%)	108 (100%)

#### Knowledge exchange networks

The knowledge networks in Burundi and DRC show multiple components, which means that the knowledge networks are disconnected and thus will inhibit scaling (Fig 1). Our data show that knowledge is being exchanged between different types of stakeholder groups (Table 5). However, for all three countries farmers and businesses are the smallest categories of organisations that knowledge is exchanged with. Somewhat surprisingly, it is not the research and training organisations that dominate, but the NGOs that make up the largest part of the composition of the knowledge networks in Burundi and Rwanda. These NGOs often operate at the international level (Table 6).

**Table 5:** Composition of the knowledge exchange networks.

	Business	Farmer	Govern-ment	NGO	Research and training	Unknown	Total	Share of collaborative network
**Burundi**	5	8	10	21	14	3	61	43%
	(8%)	(13%)	(16%)	(34%)	(23%)	(5%)	1	
**DRC**	6	7	15	18	24	7	77	28%
	(8%)	(9%)	(19%)	(23%)	(31%)	(9%)	1	
**Rwanda**	0	4	3	14	12	0	33	31%
	(0%)	(12%)	(9%)	(42%)	(36%)	(0%)	1	

**Table 6:** Number and percentage of organisations per level in the knowledge networks.

	District	Provincial	National	Supranational	Unknown	Total
**Burundi**	1	5	25	26	4	61
	(2%)	(8%)	(41%)	(43%)	(7%)	
**DRC**	10	14	11	34	8	77
	(13%)	(18%)	(14%)	(44%)	(10%)	
**Rwanda**	1	0	10	22	0	33
	(3%)	(0%)	(30%)	(67%)	(0%)	

**Fig 2:**
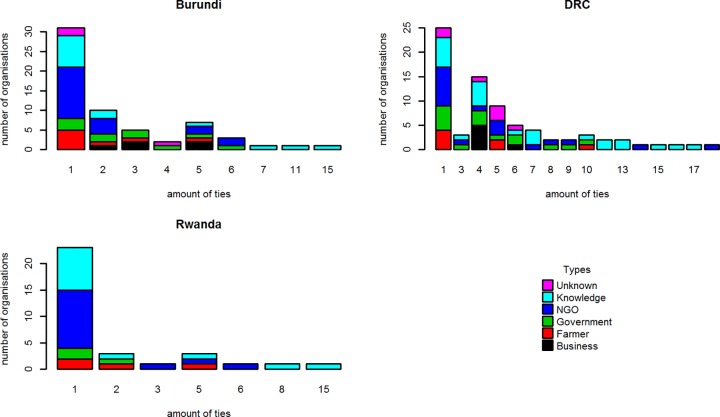
Distribution of knowledge degrees among different types of organisations.

However, even if NGOs are more present within the network, it is some of the research and training organisations that hold the most central position in the knowledge network, as they have the highest degree of the organisations within the knowledge network (Fig 2). In DRC the research and training organisations have the largest share of the knowledge network, but it is an NGO that has the highest degree. However, three research and training organisations are also among the organisations with a central position in the knowledge network in DRC.

#### Influence networks

**Fig 3:**
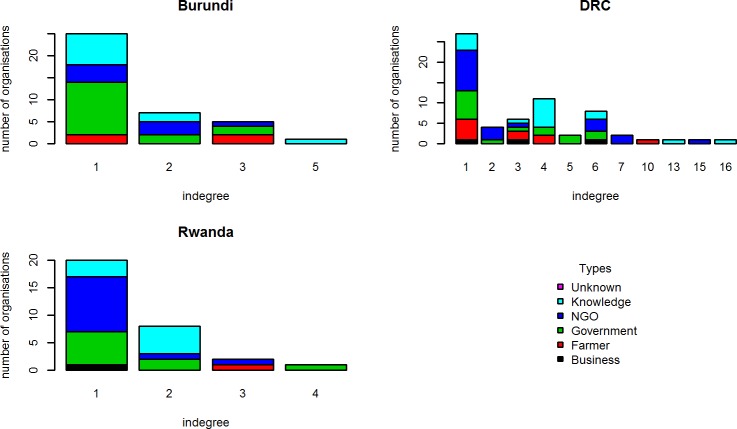
Distribution of influence indegrees among different types of organisations.

Tables 7 and 8 give an overview of the influence networks in terms of composition and administrative scale. NGO, government and research organizations are in all countries the most important source of power, although their ranking is slightly different (Fig 3). In Burundi, the government is the largest type of actor in terms of influence. In Rwanda and DRC the NGOs are the most important. Influence in these countries is thus mainly derived from legislative power (government), monetary power (government/NGOs), or knowledge (research institutes/ NGOs).

**Table 7:** Composition of the influence networks and MSPs.

	Business	Farmer	Govern-ment	NGO	Research and training	unknown	total
**Burundi**	2 (4%)	4 (9%)	16 (35%)	11 (24%)	13 (28%)	0	46
Platform members	2	0	2	4	4	0	12 (26%)
**DRC**	9 (11%)	12 (14%)	16 (19%)	24 (28%)	19 (22%)	5 (6%)	85
Platform members	1	3	3	5	2	1	15 (17%)
**Rwanda**	1 (3%)	2 (6%)	9 (26%)	13 (38%)	9 (26%)	0	34
Platform members	0	1	3	2	1	0	7 (21%)

**Table 8:** Composition of the influence networks and MSPs.

	District	Provincial	National	Supranational	Unknown	Total	
**Burundi**	1	3	18	24	0	46	32.4%
	2.2%	6.5%	39.1%	52.2%	0.0%	100.0%	
**DRC**	14	21	10	34	6	85	30.4%
	16.5%	24.7%	11.8%	40.0%	7.1%	100.0%	
**Rwanda**	3	0	12	19	0	34	31.5%
	8.8%	0.0%	35.3%	55.9%	0.0%	100.0%	

In all countries the international level is the most important in the influence network, followed by the national level. The exception is DRC where the national level is not so present, but this has to do with the fact that the MSP is organized at the provincial level (i.e. South Kivu) and not at the national level.

### Results of the ERGMs

**Fig 4:**
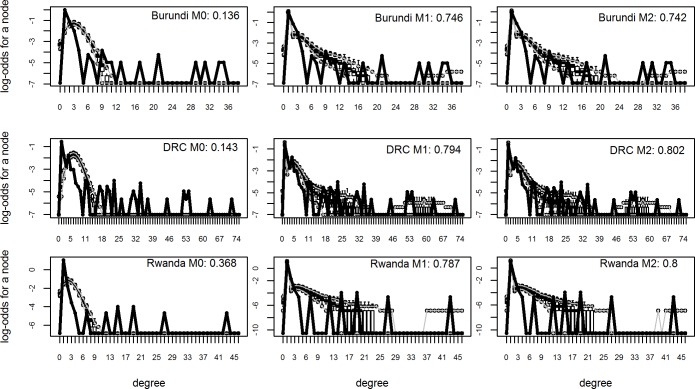
Goodness of fit over the degree distribution for different model forms.

**Fig 5:**
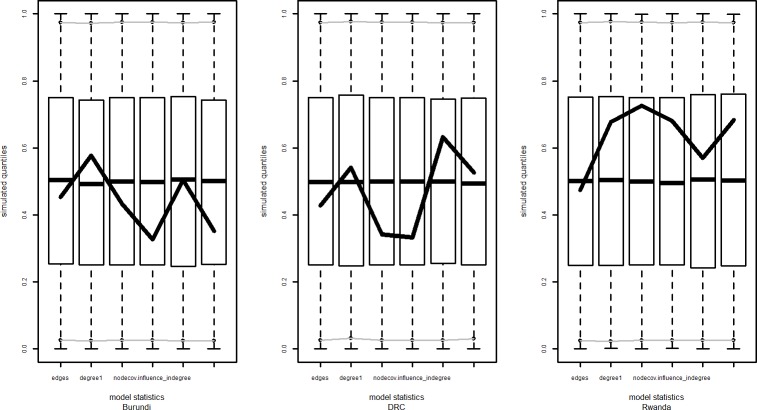
Goodness of fit diagnostics over model (M2) parameters.

Four models were tested and evaluated: starting with a simple random graph model (M0) and adding complexity in subsequent models by adding terms corresponding to our hypotheses at the node level (M1) and the dyad level (M2). Fig 4 shows the result of the goodness-of-fit percentage for the degree distribution across the 3 models. The differences between goodness-of-fit between the M1 and M2 models is very small and they lie within the same range. In order to compare the countries and test our hypotheses we have used the results of the M2 models for all three countries. Fig 5 shows the goodness-of-fit for the model parameters for these model fits.

Table 9 gives an overview of the results of the full ERGM models (M2). An overview of the ERGM results of M0 and M1 can be found in S1 Table. Regarding our first hypothesis, we find that links between the same type of organisations are positively correlated at a significant level in two of the three countries (Burundi and DRC). That means that instead of heterophily, we find a tendency for homophily (‘birds of a feather flock together’) as indicated by the positive estimates. Organisations of the same type have a 1.36 times chance to form a collaborative tie in Burundi and a 1.56 times greater chance in DRC.

**Table 9:** Exponential Random Graph Models for collaborative networks in Central Africa.

	Burundi (M2)				DRC (M2)				Rwanda (M2)			
	Estimate	Std. Error	Odds ratio	Sig.	Estimate	Std. Error	Odds ratio	Sig.	Estimate	Std. Error	Odds ratio	Sig.
Network size adjustment^a)^	-6.90				-7.02			*	-6.88			
Edges	0.87	0.32	2.39	*	0.081	0.13	32.28	*	1.57	0.56	4.82	*
Degree (1)	1.95	0.52	7.01	*	2.94	0.4	18.82	*	3.74	1.05	41.98	*
Knowledge degree	0.26	0.02	1.30	*	0.06	0.04	1.06	*	0.19	0.03	1.21	*
Influence indegree	-0.23	0.08	0.79	*	-0.03	0.03	0.98		0.08	0.08	1.08	
Administrative level	-0.54	0.18	0.59	*	-0.04	0.12	0.96		-0.42	0.19	0.66	*
Organisational type	0.30	0.14	1.36	*	0.45	0.14	1.56	*	0.16	0.12	1.17	

^a^ Network size adjustments are fixed by offset and are not estimated: pseudo-population = exp(-netsize adj.).

* Significant effect at p<0.05.

The second hypothesis regarding the effect of power and influence is not substantiated. In Burundi we find a negative estimate (-0.23) that indicates that for each additional indegree an organisation has in the influence network, it is 0.79 times less likely to form a collaborative tie. For DRC we also find such a negative estimate, although here the effect is not significant. In Burundi, influential organisations are either collaboration averse, or they are being ignored by other organisations for collaboration. In Rwanda the estimate also fall outside the cut-off rate for significance of p <0.05.

The strongest effects we find relate to the effect of the knowledge degree of organisations as indicated in hypothesis 4. We find that knowledge degree is positively correlated with the amount of ties an organisation has in the collaborative network in all three countries. This effect is strongest in Burundi where an additional degree in the knowledge network corresponds to 1.30 times the number of ties in the collaborative network. For Rwanda and DRC this effect is also positive albeit smaller (with odds ratios of 1.23 and 1.06 respectively). This confirms hypothesis 4 that knowledge exchange is significantly correlated with the amount of ties in the collaborative network.

With regard to heterophily between administrative levels that was proposed in hypothesis 5 we found significant effects for the Burundi and Rwanda MSPs. Organisations working at the same level have a smaller chance of forming a tie (indicating a higher chance for organisations at different levels to form ties).

Hypothesis 3 states that denser networks perform better with regard to scaling. Results of the ERGM helped us to calculate the densities and the mean degrees of the three networks. Because of the unknown complete network size, they could not be derived directly from the sampled ego network. However, the results of the ergm.ego were scaled to a network of a 1000 nodes for all the three countries which allowed us to compare the tendency of the organisations to form ties.

**Fig 6:**
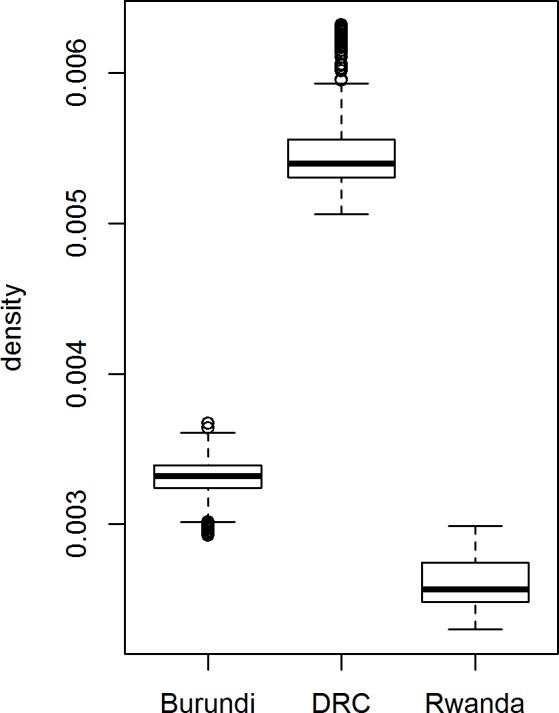
Boxplots for density based on 1000 generated networks with ERGMs for the three countries.

Fig 6 shows the three boxplots resulting from a simulation using the ergm.ego results to draw 1000 networks for a network of 1000 nodes. It shows that densities and mean degrees for Rwanda are the lowest and for DRC are the highest. Based on this result we conclude that DRC has the highest propensity to form collaborative ties and Rwanda has the lowest propensity to form a dense network. Even though it is not possible to compare these figures against an objective benchmark, we can assume that scaling in DRC will likely have the best results, compared to Burundi and Rwanda.

## Discussion

### Limitations of the study and sampling

We have conceptualized the collaborative ties between organizations as the connections through which knowledge and influence are effectuated and consequently we did not ask respondents to separately name the organisations which they collaborate with, exchange knowledge with and find influential. Instead we asked to name their collaborative partners and then choose from within this list to name the 5 organisation with whom the exchange the most knowledge, and the 5 organisations which they consider to be the most influential. The formulation of the question might lead us to exclude important knowledge, or influential organisations within the broader AIS. Furthermore, it might provide a bias towards highly connected organisations within the collaborative network with regard to knowledge and influence degrees. However, the results of the ERGM calculations (especially for the influential organisations) do not suggest this. On the contrary, we conclude that more influential organisations are less likely to form collaborative bonds in Burundi. The conclusion that organisations with a high knowledge degree are more likely to form collaborative bonds, might indeed suffer from this bias. This does provide a limitation of this study, but given the well-established link in between knowledge creation and innovation in the existing innovation literature, we think that this bias did not influence the conclusions of our study.

Another limitation of the study has to do with our decision to model the networks as ego-networks. The data sampling for the networks are based on a one-wave snowball sample and this is not exactly the same as the ego networks described by Krivitsky and Morris (66). By modelling our collaborative networks as ego networks we have essentially ignored some of the additional information we have in our sample regarding alter-alter ties that could be used to model ‘higher-order effects’ such as tendencies for triadic closure. By applying ergm.ego modelling we have assumed that the overlap between egos and alters is small enough (for Burundi 7.0%, for DRC 7.8% and for Rwanda 5.6%) to be able to ignore these triadic effects. However, it would be good to check this assumption by gathering more information on the networks of actors within AIS that are linked to the MSP but not a direct member of it in a later stage.

### Capacity to innovate in the MSP networks

Regarding the capacity to innovate we find that the absence of businesses in the collaborative network all three countries means that stakeholder representation in the networks is not proportionally balanced, which might negatively affect the capacity to innovate. The MSP may respond less to the needs of the private sectors and entrepreneurial activities which forms a core function of technological innovation networks [45, 71]. The knowledge networks are dominated by NGOs (in terms of presence) and research and extension organisations (in terms of degree centrality). The underrepresentation of farmer organisations and businesses in the knowledge exchange networks of Burundi and DRC may further exacerbate this potential weakness of the three MSPs in terms of their capacity to innovate. In other words, capacity for innovations that require a high level of knowledge exchange (e.g. local adaptation of cropping practices) is relatively weakly developed in these MSPs. A potential explanation is that the MSPs in this study prioritised removing institutional rather than technical barriers to agricultural development [16].

The results of the ERGMs showed that the collaborative networks are important conduits for knowledge exchange, as the organisations that possess complementary knowledge are more likely to be collaborated with. In contrast, the effects of influence differ from country to country.

When comparing the densities (and mean degree of collaboration) of the collaborative networks in the three countries, we observe that DRC is highest followed by Burundi and Rwanda. This means that–on average–organisations in DRC are collaborating more with other organisations than in Burundi and Rwanda. Based on our proposition, the capacity to innovate in DRC will benefit from this dense network. A potential explanation for the high mean degree in DRC is that general partnerships as well as social capital among organisations are relatively more developed. This is out of necessity since state governance systems to support farmers and other stakeholders are much weaker in DRC. Based on a study of social capital among farmers in DRC, Lambrecht et al. [72] concluded that social capital indicators do not only affect awareness, but also capacity to innovate (which they refer to as “try-out”). In Rwanda the state fulfils a much stronger governance role in the network. Burundi is a mix of both the governance models in Rwanda (with a centralised government) and DRC (where the government role is taken over by NGOs), with a mix of government and NGO influence.

Influential organisations are less likely to be collaborated with in Burundi and capacity to innovate could suffer as there may be insufficient actors who can create space to experiment and create legitimacy of new innovations.

### Scaling of innovation in the MSP networks

The structure of the collaboration networks, the knowledge networks and the influence networks can tell us about the potential of the MSP to support scaling of innovation within levels (outscaling) and across different levels (upscaling). The collaborative networks analysed in this study were dominated by supranational and national organisations (associated with the National Agricultural Research System—NARS), whereas local organisations were mostly absent. The central position of the NARS in the knowledge networks provides both opportunities and constraints for scaling of innovation. NARS and their extension systems form part of broader AIS that have the ability and infrastructure to reach many farmers and other stakeholders [73]. However, incumbent research and training systems have path-dependencies, sunk investments and a certain institutional logic, which is not easy to change and whose efficiency and innovation capacity is often low [74, 75]. The question is therefore whether the prominent placement of these types of organisations within the MSP networks will foster or hamper the removal of institutional barriers to innovation and scaling.

In all three countries, the local and provincial levels are mostly absent in the influence and knowledge networks, which might indicate poor connectivity between this level and the national level of the MSP, and vice versa. Other studies confirm that MSPs that are implemented in such linear systems will reinforce the top-down transfer of innovation paradigm, rather than foster systems approaches where innovation emerges from interactions between different types of stakeholder groups across different levels [16].

The results of the ERGMs indicate that, at least in Burundi, there is a clear tendency for organisations that operate at different levels to form a link. This will contribute positively to scaling. The results of the ERGM in Burundi show strong homophily between the same type of organisations, and heterophily when it comes to the administrative level. This indicates a scaling process in which organisations are sharing knowledge that is relevant for their type of organisation. Scaling is thus done mostly within the same type of organisation because no ‘translation’ of knowledge is necessary between organisations that use the same type of ‘institutional logic’ [76, 77].

### Recommendations for policy and further research

This paper provides a first analysis of the early stages of the MSP networks in the three countries. Continuous mapping of MSP networks over time will enable a longitudinal analysis of network evolution and also link it to the actual performance of the MSP with regard to achieving development impacts.

Based on the results of this study we can make some recommendations for the MSPs in the three countries based on their current structural characteristics and deficiencies, combined with insights in the underlying processes that are likely to have influenced the networks formation. Such insights could be used proactively to think about innovation network ‘architecture’ or ‘building’ to achieve specific types of innovations, innovation processes or scaling pathways [20, 78, 79]. In all countries this recommendation has to do with the inclusion of more farmer and business representatives within the MSP, to ensure that innovation and scaling is more end-user inclusive. For DRC and Burundi more attention to developing the knowledge exchange network is necessary in order to connect the different parts of the network (across hierarchies and spatial scales).

## Conclusions

In this paper we have explored the potential for innovation and for scaling of innovations of three MSPs for agricultural research and development in Rwanda, DRC and Burundi. A series of propositions and hypotheses that are based on innovation and scaling literature have guided us in comparing the collaborative, knowledge and influence networks and functions associated with these MSPs in contrasting governance contexts.

With regard to the capacity to innovate we observed that all three MSP networks are dominated by NGOs with an apparent lack of involvement of the business sector. The dominance of development organisations and lack of entrepreneurial capacity in these networks may hinder social learning and the development of innovations that are commercial and respond to end-user needs. Knowledge plays an important role in the innovation network and the amount of knowledge exchange is positively correlated with the amount of collaborative ties an organisation has within the innovation network. In DRC and Burundi the decentralised governance structure seems to create a problem in that MSPs are not strongly linked to the most influential agencies, which could negatively affect their legitimacy and create obstacles for achieving institutional (policy) innovations and upscaling for impact.

The MSP networks are dominated by supranational and national organisations, whereas local organisations were mostly absent. Such networks are thus less geared towards the outscaling of knowledge intensive innovation and their local adaptation to diverse end-users and environments. The study illustrates that MSP networks are diverse and context-specific. We propose that MSPs should not be used as blueprint vehicle for supporting innovation and scaling, but that more research is required to understand how the institutional setting (e.g. governance) and under-representation of certain actors (e.g. private sector) affect the ability of MSPs to stimulate capacity to innovate and achieving development impact at scale.

## Supporting information

S1 File (ZIP)

S2 File (ZIP)

S1 R-scripts (R)

S1 Table (DOCX)
