# Supplementary figures and images for: Social network analysis of multi-stakeholder platforms in agricultural research for development: Opportunities and constraints for innovation and scaling
Source: PLoS One. 2017 Feb 6;12(2):e0169634. doi: 10.1371/journal.pone.0169634 (PMC5293196; doi:10.1371/journal.pone.0169634)

# Sample statistics

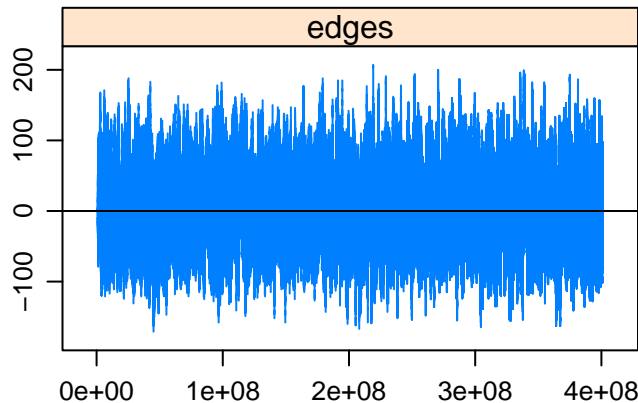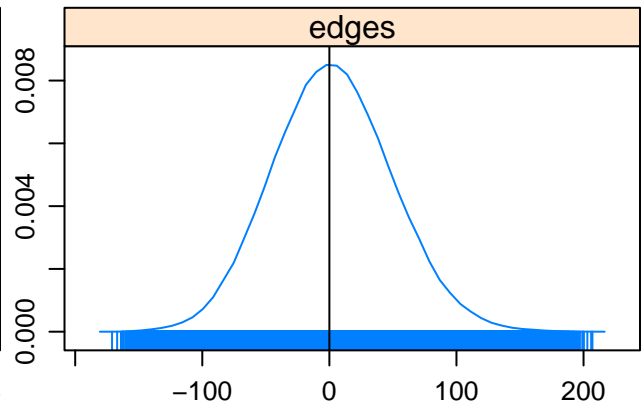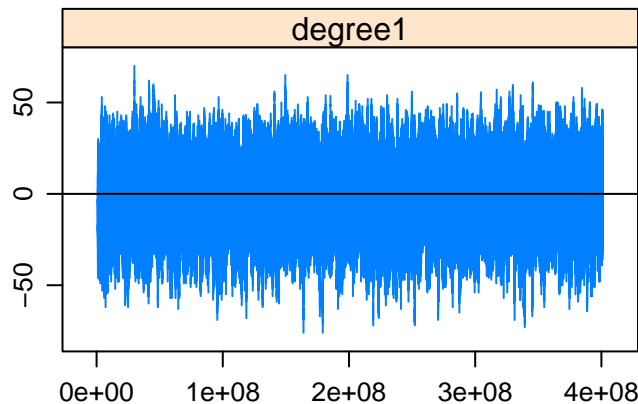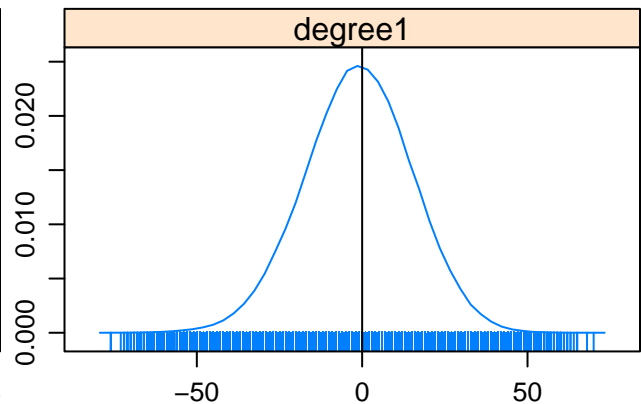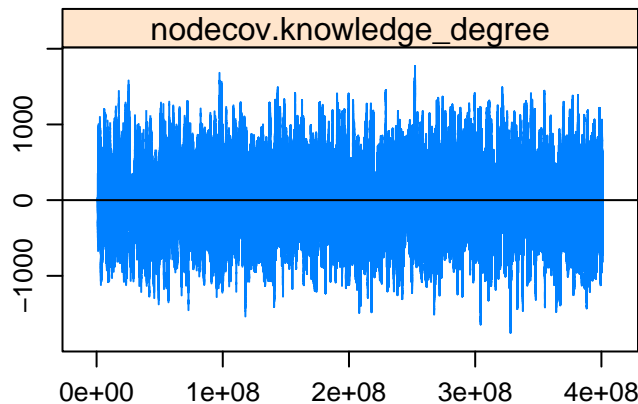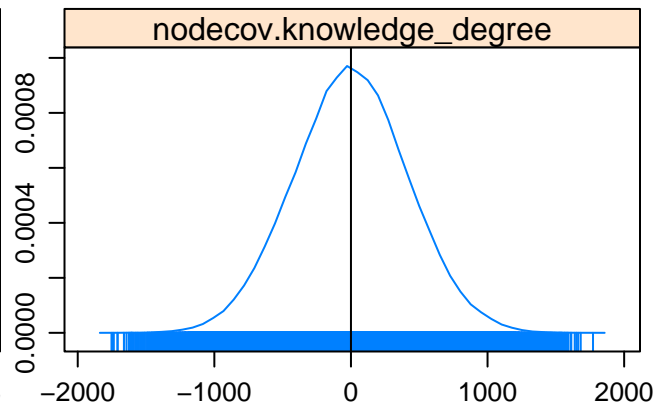

# Sample statistics

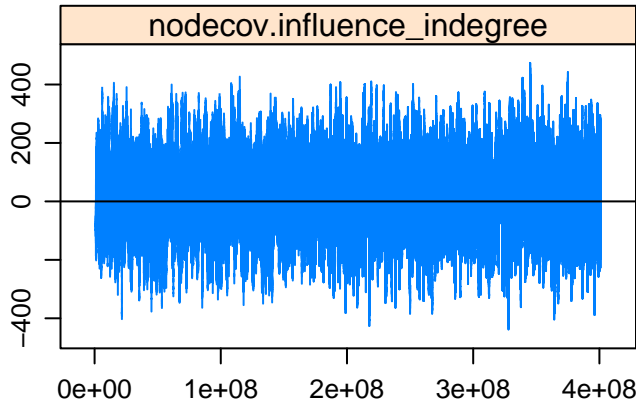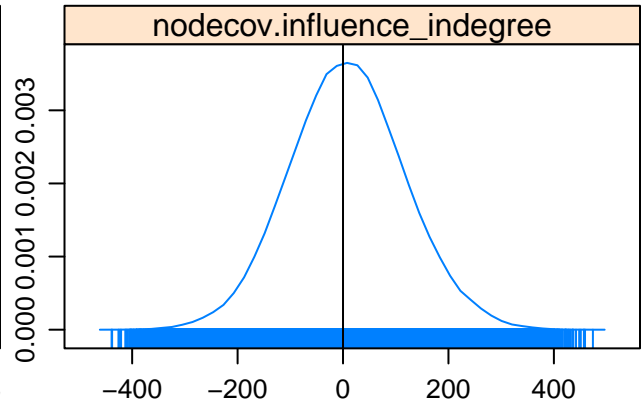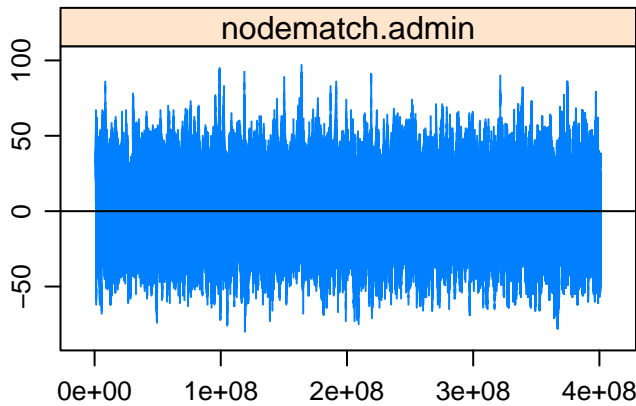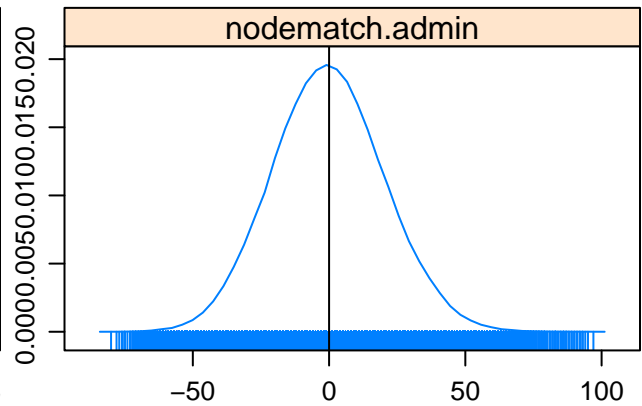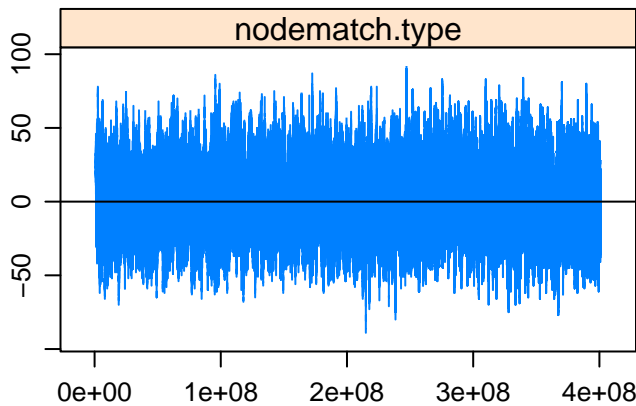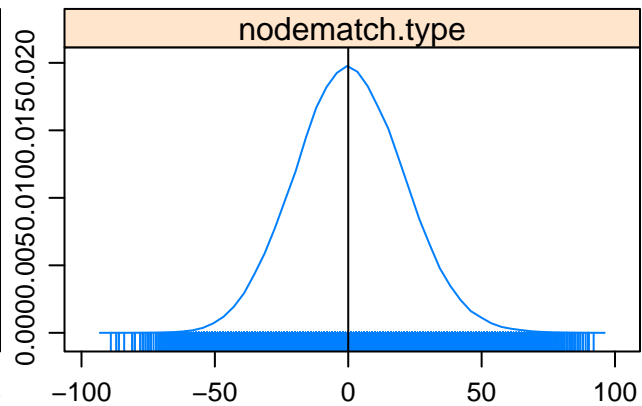

Supplement: S2 File — (ZIP) [file pone.0169634.s002.zip › Rmix1_ego diagnostics.pdf]

# Sample statistics

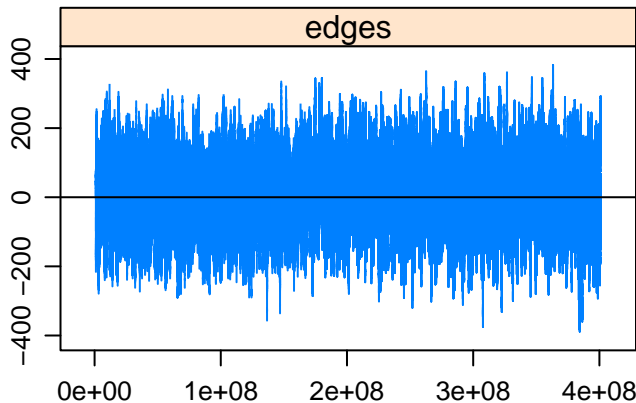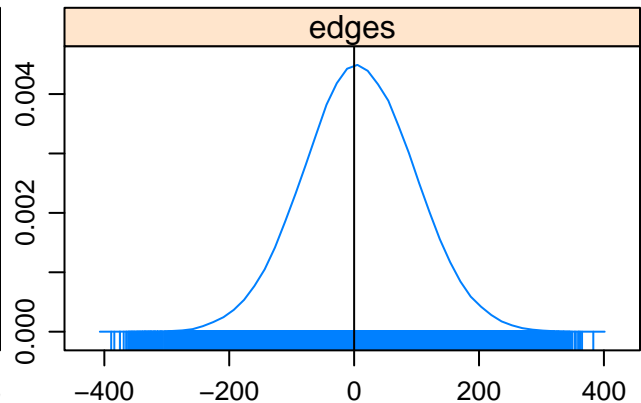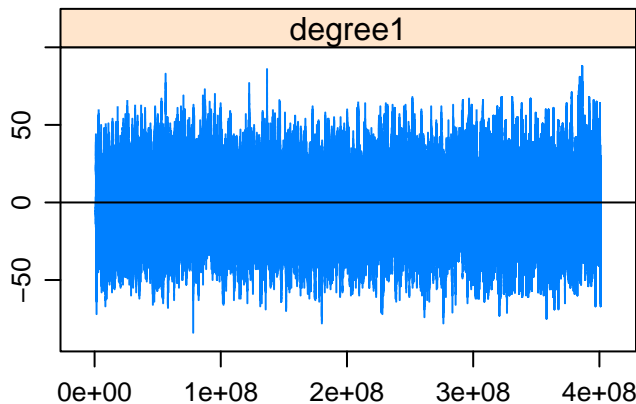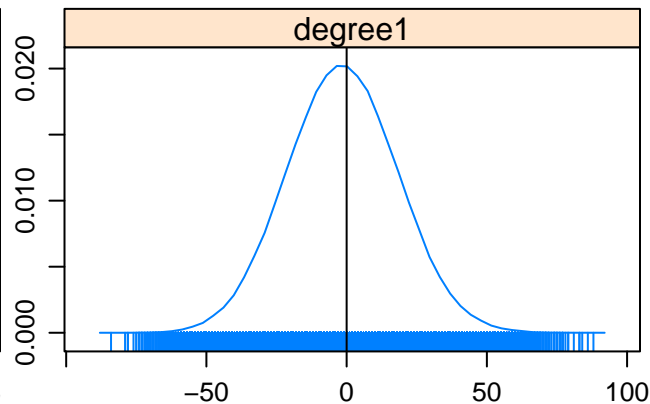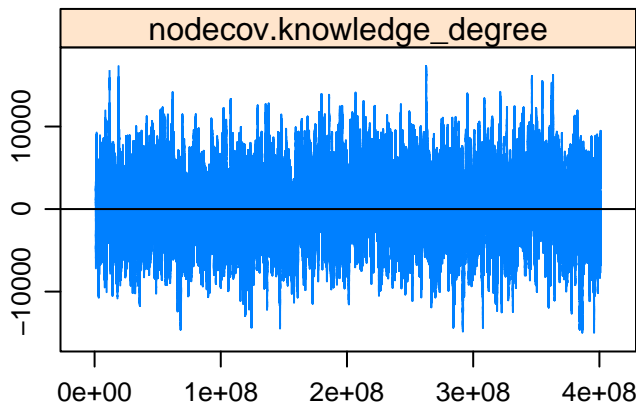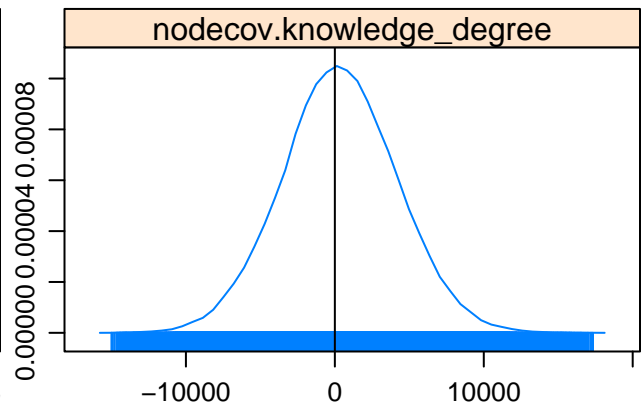

# Sample statistics

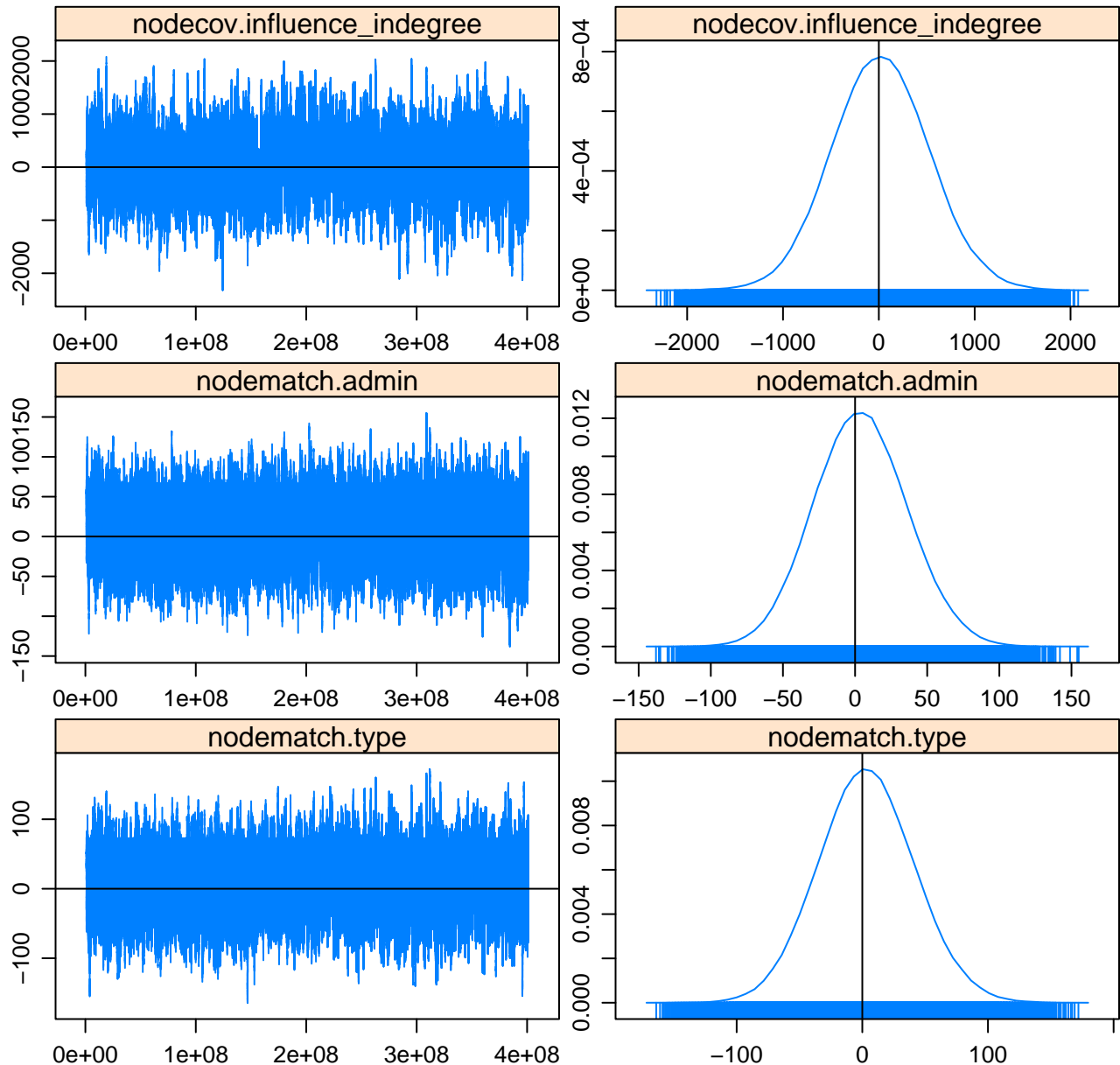

Supplement: S2 File — (ZIP) [file pone.0169634.s002.zip › Dmix1_ego diagnostics.pdf]

# Sample statistics

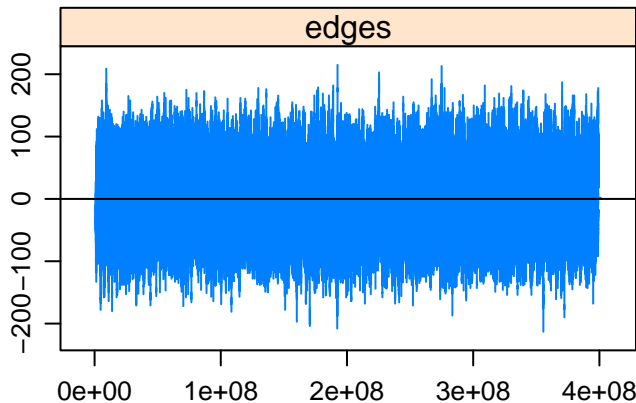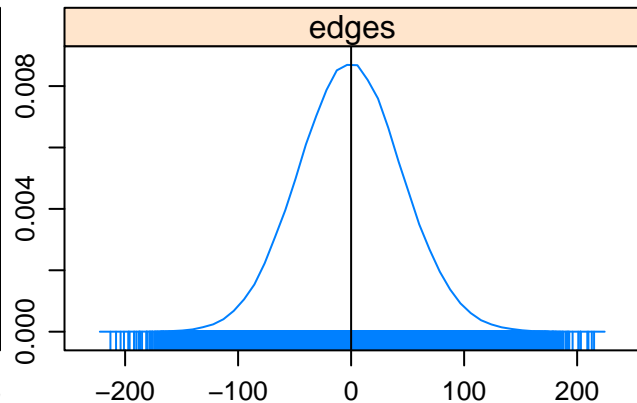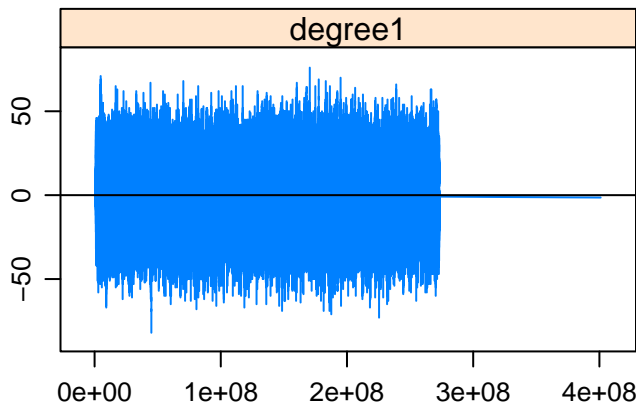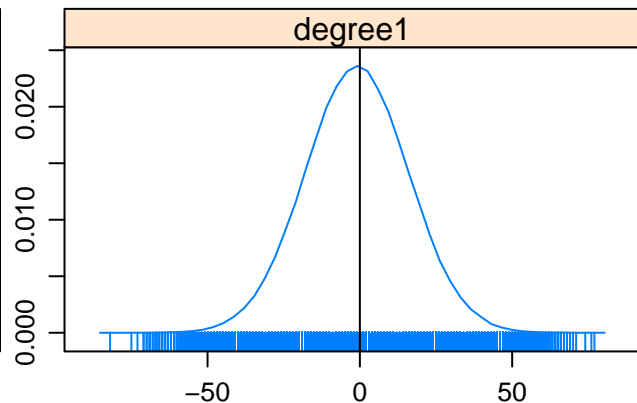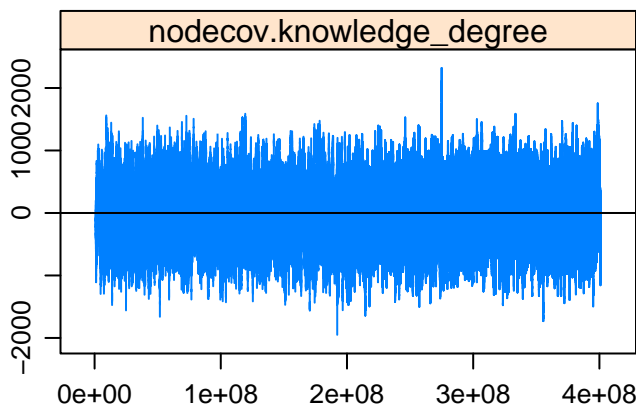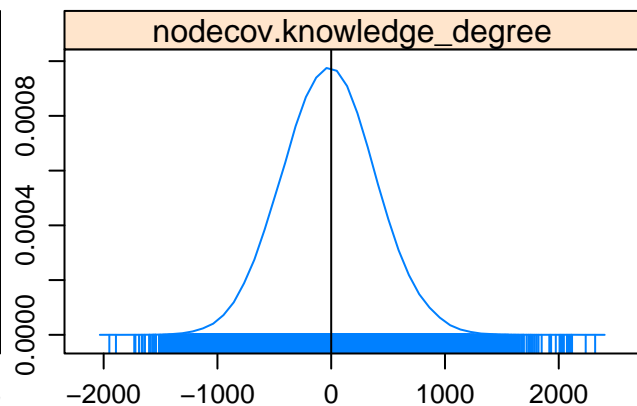

# Sample statistics

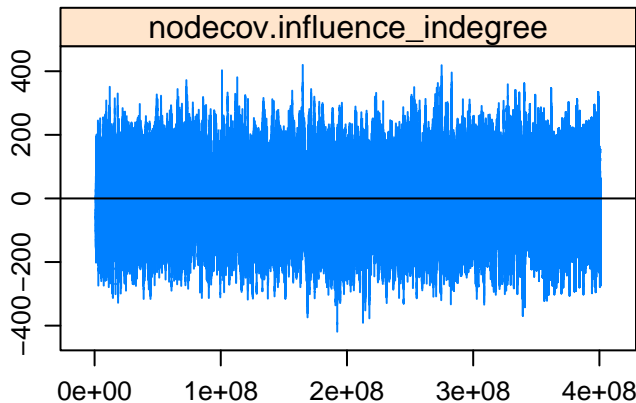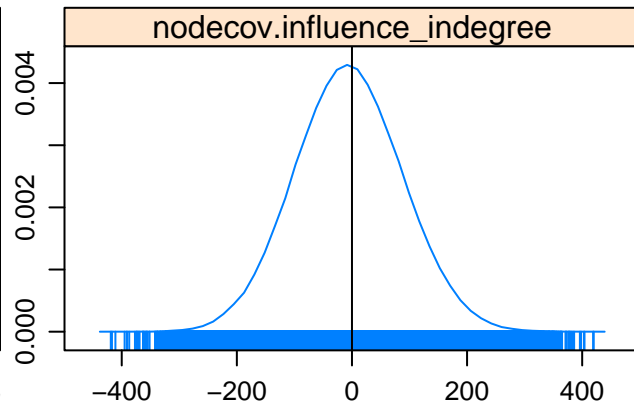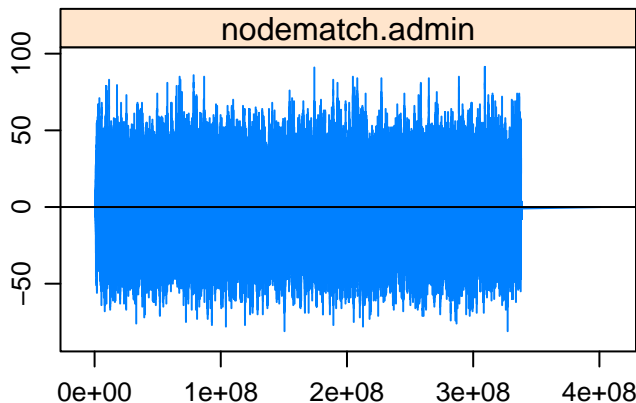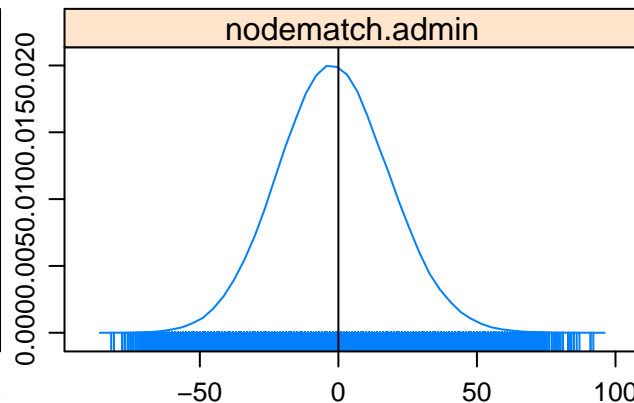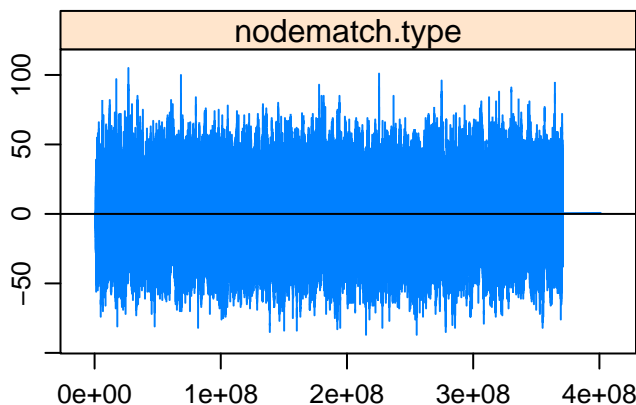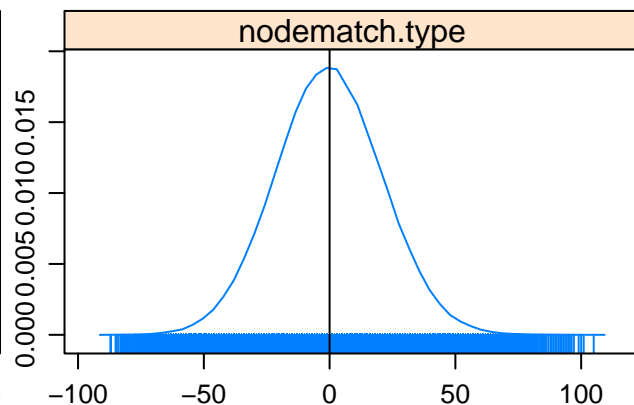

Supplement: S2 File — (ZIP) [file pone.0169634.s002.zip › Bmix1_ego diagnostics.pdf]
