# Supplementary material for: Social network analysis of multi-stakeholder platforms in agricultural research for development: Opportunities and constraints for innovation and scaling
Source: PLoS One. 2017 Feb 6;12(2):e0169634. doi: 10.1371/journal.pone.0169634 (PMC5293196; doi:10.1371/journal.pone.0169634)
Supplement: S1 Table — (DOCX) [file pone.0169634.s004.docx]

**S4 Table overview of ERGM results for all models**

|  | **M0** | | | | **M1** | | | | **M2** | | | |
| --- | --- | --- | --- | --- | --- | --- | --- | --- | --- | --- | --- | --- |
| **BURUNDI** | Estimate | Std. Error | OR |  | Estimate | Std. Error | OR |  | Estimate | Std. Error | OR |  |
| netsize.adj | -6.90 |  |  |  | -6.90 |  |  |  | -6.90 |  |  |  |
| edges | 1.21 | 0.15 | 3.35 | * | 0.84 | 0.32 | 2.32 | * | 0.87 | 0.32 | 2.39 | * |
| degree1 |  |  |  |  | 1.97 | 0.53 | 7.18 | * | 1.95 | 0.52 | 7.01 | * |
| Knowledge degree |  |  |  |  | 0.26 | 0.02 | 1.29 | * | 0.26 | 0.02 | 1.30 | * |
| Influence indegree |  |  |  |  | -0.23 | 0.07 | 0.79 | * | -0.23 | 0.08 | 0.79 | * |
| Administrative level |  |  |  |  |  |  |  |  | -0.54 | 0.18 | 0.59 | * |
| Organisational type |  |  |  |  |  |  |  |  | 0.30 | 0.14 | 1.36 | * |
|  |  |  |  |  |  |  |  |  |  |  |  |  |
| **RWANDA** | Estimate | Std. Error | OR |  | Estimate | Std. Error | OR |  | Estimate | Std. Error | OR |  |
| netsize.adj | -6.88 |  |  |  | -6.88 |  |  |  | -6.88 |  |  |  |
| Edges | 0.96 | 0.22 | 2.62 | * | 1.46 | 0.56 | 4.30 | * | 1.57 | 0.56 | 4.82 | * |
| degree1 |  |  |  |  | 3.71 | 1.03 | 40.91 | * | 3.74 | 1.05 | 41.98 | * |
| Knowledge degree |  |  |  |  | 0.19 | 0.03 | 1.21 | * | 0.19 | 0.03 | 1.21 | * |
| Influence indegree |  |  |  |  | 0.08 | 0.09 | 1.08 |  | 0.076 | 0.08 | 1.08 |  |
| Administrative level |  |  |  |  |  |  |  |  | -0.42 | 0.19 | 0.66 | * |
| Organisational type |  |  |  |  |  |  |  |  | 0.16 | 0.12 | 1.17 |  |
|  |  |  |  |  |  |  |  |  |  |  |  |  |
| **DRC** | Estimate | Std. Error | OR |  | Estimate | Std. Error | OR |  | Estimate | Std. Error | OR |  |
| netsize.adj | -7.02 |  |  |  | -7.02 |  |  |  | -7.02 |  |  |  |
| edges | 1.87 | 0.10 | 6.48 | * | 0.91 | 0.12 | 2.48 | * | 0.82 | 0.13 | 2.28 | * |
| degree1 |  |  |  |  | 2.94 | 0.39 | 18.98 | * | 2.94 | 0.4 | 18.82 | * |
| Knowledge degree |  |  |  |  | 0.06 | 0.00 | 1.06 | * | 0.06 | 0.04 | 1.06 | * |
| Influence indegree |  |  |  |  | -0.02 | 0.03 | 0.98 |  | -0.03 | 0.03 | 0.98 |  |
| Administrative level |  |  |  |  |  |  |  |  | -0.04 | 0.12 | 0.96 |  |
| Organisational type |  |  |  |  |  |  |  |  | 0.45 | 0.14 | 1.56 | * |

*) Significant effect at p<0.05
